# Factors Associated with Refraining from Health Checkups during the COVID-19 Pandemic in Japan

**DOI:** 10.3390/healthcare11172385

**Published:** 2023-08-24

**Authors:** Naoko Ito, Hiroki Sugimori, Takeshi Odajima, Naohito Yoshimura, Shigeki Muto, Maki Hirao, Mika Ninohei, Takeo Nakayama

**Affiliations:** 1 Department of Nursing, Faculty of Sports and Health Science, Daito Bunka University, Saitama 355-8501, Japan; nito@ic.daito.ac.jp (N.I.);; 2 Japanese Red Cross Kanto-Koshinetsu Block Blood Center, Tokyo 153-0053, Japan; 3 Department of Nursing, Faculty of Nursing, Iryo Sosei University, Iwaki 970-8551, Japan; 4 Seirei Social Welfare Community’s Health Care Division, Hamamatsu 430-0906, Japan; smuto@sis.seirei.or.jp; 5 Department of Health Science, Faculty of Sports and Health Science, Daito Bunka University, Saitama 355-8501, Japan; mhirao@ic.daito.ac.jp; 6 Department of Health Informatics, School of Public Health, Kyoto University, Kyoto 606-8501, Japan

**Keywords:** health checkups, refrain from health checkups, COVID-19, epidemiology

## Abstract

This study aimed to determine the characteristics of people who refrained from having regular checkups due to the spread of the novel coronavirus 2019 (COVID-19) infection and the factors associated with this behavior. We conducted a nationwide internet survey of 4593 males and females aged 20–69 in Japan regarding their health checkups from April 2020 to March 2021, when COVID-19 was widespread. Individuals who received checkups during this time were “the receiving group”; those who did not were “the refraining group”. Personal attributes, responses to a health questionnaire and other items were used to compare the groups. The analysis showed that males over 53 refrained from having health checkups compared to those younger. On the other hand, males with higher personal incomes who never skipped breakfast received health checkups. Females with children under 18 years were less likely than those without to receive health checkups. For males, the characteristic factors were economic and health awareness and literacy. Females were less aware of medical checkups. Moreover, they demonstrated an inability to maintain an everyday rhythm. No factors were common to males and females, indicating the need to consider separate strategies for encouraging males and females to obtain annual health checkups.

## 1. Introduction

Regular health checkups are essential for monitoring health status. In Japan, health check-ups are available to almost all segments of the population throughout their life course [1]. There are legally required health check-ups such as health check-ups for infants and preschool children, an annual health check-up for school children, an annual health check-up for full-time employees, an annual stress test for employees, and an annual specific health check-up for people aged between 40 and 74 to prevent lifestyle-related diseases [2]. Workers are obliged to undergo health checkups once a year under the Industrial Safety and Health Act and are recommended to undergo health checkups, and in principle cannot refuse to undergo health checkups. The employer pays all the costs of these periodic health checkups [3]. It is mandatory to have an annual health checkup at the workplace or by the local government.

In March 2020, the World Health Organization declared the new coronavirus 2019 (COVID-19) infections a pandemic. In Japan, the first state of emergency was declared in April 2020. The state of emergency introduced various behavioral restrictions to prevent the spread of infection, such as refraining from unnecessary outings [4]. Visiting medical institutions and shopping for daily necessities were not considered “unnecessary outings”. However, the number of people visiting medical institutions decreased compared to the number that did so during the pre-outbreak period, indicating that people were refraining from going to medical institutions. Igarashi conducted a survey using a web panel on the status of visits to medical institutions from 2020 to 2021. They reported that 25.7% of regular and 28.7% of brief visits were less frequent than the previous year [5]. The rate of medical checkups was also severely affected by the COVID-19 pandemic, with the rate of specified medical checkups falling for the first time since the system began in 2008: the rate in 2020 was reported to be 53.4%, down 2.2 percentage points from the previous year [6]. To prevent COVID-19 infection, many people refrained from or delayed undergoing health checkups. This tendency may have delayed a medical response to lifestyle-related diseases and illnesses.

A study of factors associated with postponing medical examinations during the COVID-19 pandemic in Germany has been reported. People who postponed their checkups because of the COVID-19 pandemic were associated with the presence of chronic disease [7]. The leading cause for postponement was fear of contracting COVID-19 and its potential health hazards. This anxiety is particularly pronounced among those with chronic diseases. In Japan, reports have been published on the status of medical visits by the department [8] and the rate of cancer care visits due to the effects of COVID-19 [9]. However, only reports on dental examinations have examined the trends and characteristics of people who refrained from seeking medical care [10,11].

The spread of infectious diseases has affected access to medical facilities and health checkups. Related organizations and medical care providers are taking various measures to address this problem. Historically, many establishments conducted health checkups using a face-to-face or group method. The date, time, and place were predetermined, and many people were invited to the same site for the examination. Following the COVID-19 pandemic, a protocol suitable for the “new way of life” was discussed. In May 2020, eight related organizations proposed a joint manual on COVID-19 countermeasures for health checkups. In addition, a guideline for each sector that voluntarily summarizes appropriate infection prevention measures for each industry was formulated [12]. However, operational issues in actual workplaces had yet to be finalized. In 2019, 79.0% of employees were covered by employee insurance, while National Health Insurance covered 34.9% [13]. The health checkup rate varies depending on the type of health insurance. The consultation rate is extremely low among people with National Health Insurance, as they have difficulty gaining opportunities for health checkups. People with National Health Insurance include unemployed individuals and those who cannot be examined at their workplaces.

However, the previous studies focused on actual (non)-attendance rather than postponed health checkups, not yet identifying the trends and characteristics of individuals who refrained from undergoing health checkups. It is possible that targeted measures have not been implemented for those who did not take action to receive health checkups.

Therefore, this study aimed to understand why individuals refrained from going for health checkups during the COVID-19 pandemic.

## 2. Methods

### 2.1. Participants and Data Collection

The survey targeted participants (approximately 1.2 million in June 2020) registered with a research company (Macromill, Inc., Tokyo, Japan) specializing in web-based surveys. The target sample size was 4000 respondents (2000 males and females) between the ages of 20 and 69 years, whom the research company randomly matched and allocated to ensure that the same sampling ratios for those who “received” and “did not receive” health checkups in 2020 and for “employee insurance” and “National Health Insurance” membership. The survey was conducted over five days from 4 to 8 November 2021 and was closed when the number of respondents exceeded the target sample size.

### 2.2. Instruments and Variables

#### 2.2.1. Health Checkups

Health checkups in this study referred to physical examinations. Health checkups include a medical interview, physical measurements (height, weight, and abdominal circumference), blood pressure measurement, blood test, and urinalysis [1]. They did not include cancer screening, prenatal or dental checkups, or examinations performed to receive medical treatment at hospitals. Respondents were asked about the status of their health checkups during the period from April 2020 to March 2021, when COVID-19 was widespread, using the following four options: “I had a checkup as usual”, “I refrained from having a checkup but took it at a later time”, “I refrained from having a checkup but will have one in the future”, and “I refrained from having a checkup but will not have one in the future”.

#### 2.2.2. Participants’ Characteristics and Health Status

Personal characteristics included the following variables: sex, age, marital status, presence of children under 18 years, employment status, annual income, and education level attained.

In preparation for the statistical analysis, age was grouped into above- and below-median groups. Marital status was divided into single and married. The single group included participants who were separated or widowed. Participants who earned income from some type of employment, such as public servants, company employees, self-employed, freelance, and part-time workers, were defined as having an occupation, while those who were housewives (or househusbands), students, and unemployed were defined as not having an occupation. Individual annual income was used for analysis instead of household income to grasp differences in medical examination behavior based on individual income. The group was split with a cutoff value of 6 million yen. We used university versus graduate school as the cutoff for grouping by education level.

For specific health-related items, we used interview questions included in checkups and other medical examinations to understand participants’ physical condition and daily habits better [14]. The questionnaire included the status of medications related to an underlying disease; daily habits such as exercise, diet, drinking, and smoking; weight gain or loss and willingness to improve lifestyle habits. Participants were asked the following as examples: “Do you skip breakfast more than 3 times a week?” (response options: yes; no), “In your daily life, do you walk or do any equivalent amount of physical activity for more than 1 h a day?” (response options: yes; no), “Are you in the habit of doing exercise to sweat lightly for over 30 min at a time, 2 times weekly, for over a year?” (response options: yes; no), “How often do you drink (sake, shochu, beer, wine, whisky, brandy, etc.)?” (response options: everyday; sometimes; rarely drink), “Have you gained or lost more than ±3 kg in the past year?” (response options: yes; no).

Respondents who answered that they did not intend to improve their lifestyle habits were considered to have “no desire to improve”. In contrast, other answers were considered to have a “desire to improve” as the participants intended to do so.

### 2.3. Statistical Analysis

FY2020 (April 2020 to March 2021), participants who received checkups as in previous years and those who refrained but received them later were classified in the receiving group (HC+). Participants who refrained from having the checkup but intended to have it in the future and those who refrained with no intention of having it in the future were classified in the refraining group (HC−).

Chi-square tests were performed for each item. We stratified the study cohort by sex. Then, we conducted the unconditional multivariate logistic regression analysis with the examination group as the objective variable and the factors that showed significant differences in the chi-square test as explanatory variables. To further improve the accuracy of the regression analysis model, we conducted the stepwised multivariate logistic regression analysis to calculate adjusted odds ratios and 95% confidence intervals.

SAS version 9.4 (SAS Institute Inc., Cary, NC, USA) was used for all statistical analyses. The statistical significance level was set at less than 5%.

### 2.4. Ethical Considerations

Survey participants are registered with Macromill, Inc. Registration entails explicit consent to the use of their survey data. Macromill, Inc. protects personal information.

This study was conducted after the Ethics Review Committee for Life Science and Medical Research Involving Human Subjects, Daito Bunka University, approved its design (approval number DHR21-008).

## 3. Results

Data were analyzed for 4593 participants: 2289 were male, and 2304 were female, with a mean age of 47.6 ± 12.86 years. 2312 (50.3%) males and females received health checkups in 2020; 2281 (49.7%) males and females did not receive them.

### 3.1. Relationship between Health Checkup Status and Individual Characteristics

Table 1 shows the results of comparing each item in the refraining and receiving groups by sex. The chi-square test results showed that compared to those in the receiving group, a significantly higher proportion of males in the refraining group were 53 years and older (50.8%), single (56.1%), had no children under 18 years (45.4%), were unemployed (54.4%), and had an annual personal income of under 6 million yen (51.8%). In contrast, compared to receiving-group females, a significantly higher proportion of those in the refraining group were married (53.6%), had children under 18 years (57.4%), were unemployed (64.1%), had an annual personal income under 6 million yen (50.4%), and had an educational level of junior high, high, or vocational school (52.5%).

Table 2 compares males and females in both groups regarding health status and lifestyle items. The chi-square test results showed that more than half the males who refrained from going for their checkups took no medication for hypertension or hyperlipidemia, skipped breakfast, did not engage in physical activity or exercise, had no desire to improve their lifestyle, and smoked. Females who did not take medication for hypertension or hyperlipidemia, skipped breakfast, did not engage in physical activity or exercise, walked slowly, and gained or lost weight were significantly more likely to refrain from seeing a doctor.

### 3.2. Multivariate Analysis

Table 3 and Table 4 show the results of the multivariate logistic regression analysis stratified by sex, using the factors for which significant differences were found in the chi-square test as explanatory variables. In addition, the stepwised regression analysis was performed.

Individuals 53 or older refrained from having health checkups compared to younger people (low = 1.00) (OR, 0.54; 95% CI, 0.41–0.71). Those not taking medication for hypertension (OR, 0.71; 95% CI, 0.52–0.97) who were physically inactive (OR, 0.75; 95% CI, 0.58–0.97) also refrained from health checkups. On the other hand, males with higher personal income (OR, 1.80; 95% CI, 1.37–2.37) who never skip breakfast received health checkups (OR, 1.73; 95% CI, 1.25–2.39).

Females with children under 18 years were less likely than those without (No = 1.00) to have health checkups (OR, 0.66; 95% CI, 0.51–0.86). Those who did not exercise also refrained from having health checkups (OR, 0.68; 95% CI, 0.48–0.95). On the other hand, those who were working (OR, 3.02; 95% CI, 2.35–3.88) and had no weight gain or loss had health checkups (OR, 1.41; 95% CI, 1.09–1.83).

## 4. Discussion

We examined the factors associated with people who refrained from receiving health checkups. Our analyses revealed that the items significantly associated with health checkup status for both males and females were marital status, presence of children under 18, occupational status, and personal annual income. However, the significant items showed inverse associations between males and females. Unmarried males without children under the age of 18 refrained from having health checkups, as did females who were married and had children under 18. This finding suggests that the family backgrounds of those who refrained from health checkups differed between the sexes. A previous study reported that males should have someone close to them who encourages them to have health checkups [15,16].

Unemployed males and females and those whose annual personal incomes were low were less likely to go for medical examinations. According to previous surveys, the health checkup rate among low-income individuals is significantly less than that of high-income individuals. In fact, 42.9% of males in low-income households refrain from receiving checkups. In contrast, only 16.1% of males in high-income households do the same [17]. In the current study, individuals with low incomes were less likely to receive health checkups, indicating that economic factors are associated with this tendency.

Several health-related items were significantly associated with health checkup status: no medication for underlying hypertension or hyperlipidemia, sometimes skipping breakfast and engaging in neither physical activity nor light exercise. Similar trends were observed for both males and females. Factors influencing those who skip breakfast remain unknown. A Ministry of Health, Labour, and Welfare survey found that 15.5% of males and 9.1% of females skip breakfast [18]. The current study’s findings indicate that a higher than average percentage of males and females skip breakfast: 538 males (23.5%) and 543 females (23.6%) skipped breakfast, suggesting that daily life rhythm may have changed due to the COVID-19 pandemic.

Males who did not engage in physical activity or light exercise, and those not motivated to improve their lifestyle, were less likely to receive checkups, as were females who did not engage in physical activity or exercise. In Japan, before the COVID-19 pandemic, according to the OECD reports, the rate of health checkups among people with unhealthy lifestyles and risk factors such as smoking, lack of exercise, and high blood pressure was lower than among those with healthy lifestyles [1]. Our analysis is consistent with this data, revealing that individuals lacking physical activity or exercise were also less likely to undergo medical examinations. This relationship suggests that social conditions requiring people to avoid going out during the pandemic may have further reinforced unhealthy lifestyle tendencies. Therefore, adopting physical activity and exercise in one’s daily life—even indoors—and maintaining an awareness of one’s health may lead to more frequent health checkups.

According to the regression analysis, items significantly associated with medical checkup status for males were age 53 or older, annual personal income under 6 million yen, not taking hypertension medication, and skipping breakfast. Since income and the incidence of hypertension generally increase with age, we infer that the true relationship between the items is low. Males over 53 years old were more health-conscious and therefore did not see a doctor for fear of contracting COVID-19. A study examining factors associated with medical examination postponement during the COVID-19 epidemic in Germany reported that people with chronic diseases avoided medical examinations for fear of health hazards [7]. Therefore, health-conscious individuals may have engaged in protective behavior, as did those with these tendencies. Our study found that males with lower personal annual incomes did not go for checkups. In previous studies, many males who did not have health checkups reported that low household income [1], suggesting a strong association between economic factors and health checkups.

Among females surveyed, those who have children under 18 years, have no occupation, do not exercise, and have gained or lost weight were more likely to refrain from seeing a doctor. The 2019 National Survey on Health and Medical Examinations, conducted before the COVID-19 pandemic, reported that the proportion of females in all age groups was higher than that of males, and the percentage of females who have not had health checkups is higher than males in all age groups, especially among females aged 30–39 years. Moreover, about 40% of them did not undergo health checkups [19]. Females with similar characteristics in the present study were also less likely to receive health checkups. This result suggests that supportive measures for daily life activities that allow females to have more spare time may facilitate their receiving health checkups.

The univariate and multivariate analysis results also showed that females who were not working were less likely to obtain health checkups. Imoto et al. reported that half (51.7%) of the females in their study who had not had a medical examination were unemployed [15]. Employed females are required by their workplaces to undergo health checkups. However, unemployed females cannot be directly encouraged to do so. This situation further depresses the health checkup rate when people are asked to refrain from going out. On the other hand, many women who are working are employed on a part-time basis, making it difficult for them to receive health checkups at their workplaces, so it is desirable for them to receive checkups as dependents [20].

However, it has long been pointed out that the medical checkup rate for female dependents of public medical insurance enrollees is low. In a study of the factors that lead to the health checkup behavior of female dependents, respondents indicated that they would like to receive health checkups at a familiar place, and those with small children would be more likely to receive checkups if temporary childcare services were available. Those working part-time would hesitate to apply for time off because of the checkups, etc. The report also states that it is necessary to respond to women’s life stages, including childcare, work, etc. [21]. Therefore, in order to improve the rate of health checkups, it was considered essential to devise a method of conducting health checkups without having to go out of the house, as well as to devise time support measures and appropriate recommendations for health checkups. To improve medical checkup rates, providing time-sensitive support measures, appropriate recommendations, and methods of conducting checkups that do not require leaving home are essential.

Recently, the Ministry of Health, Labour and Welfare in Japan has called for a shift to a “new lifestyle” to prevent the spread of infectious diseases. Practical examples include the use of mail order and electronic payment for shopping, telework, and online meetings [22]. The medical field is utilizing “online/telephone medical care and drug administration guidance” for interested patients, at least under certain conditions [23]. In the future, people may be able to have appropriate health checkups at home via the Internet, even in conditions of lockdown or confinement. Further research is essential to finding ways to improve the future rate of health examinations.

The current study had some limitations. The study was a cross-sectional survey at a single point in time. As the epidemic situation changed, awareness of COVID-19 may have changed accordingly. The study population was limited to internet users who registered with a research company for monitoring. However, the data are valuable. They represent a nationwide, broad-based survey of individual factors describing who refrained from receiving health checkups during the COVID-19 pandemic.

## 5. Conclusions

In this study, a nationwide web-based survey was administered to 4593 males and females aged 20–69 years. The survey took place during the COVID-19 pandemic in Japan. It examined factors associated with individual tendencies to avoid health checkups. The results showed that economic factors and health literacy and self-awareness were characteristic factors for male, while female have under children 18 years, have no occupation, do not exercise, and “have gained or lost weight. For females, factors related to child rearing and employment were considered to be the lack of a daily rhythm. Thus, methods of recommending health checkups that focus on the different characteristics of males and females must be explored. This consideration should include separate health checkup recommendations for males and females. Furthermore, home-based health checkups should be made readily available.

## Figures and Tables

**Table 1:** Comparison of characteristics in the refrained-from-health-checkups and health-checkups groups by sex.

	Males (n = 2289)						Females (n = 2304)					
Variable	HC−		HC+				HC−		HC+			
	n	(%)	n	(%)	χ^2^	*p* Value *	n	(%)	n	(%)	χ^2^	*p* Value *
Age ^a^					5.55	0.019					0.32	0.569
Low	363	(45.6)	433	(54.4)			704	(50.8)	681	(49.2)		
High	758	(50.8)	735	(49.2)			456	(49.6)	463	(50.4)		
Marital status					33.54	<0.001					15.64	<0.001
Single ^b^	535	(56.1)	418	(43.9)			393	(45.1)	479	(54.9)		
Married	586	(43.9)	750	(56.1)			767	(53.6)	665	(46.4)		
Children under 18 years					3.95	0.047					15.54	<0.001
No	287	(45.4)	345	(54.6)			248	(46.3)	288	(53.7)		
Yes	224	(39.7)	340	(60.3)			425	(57.4)	315	(42.6)		
Occupation					107.89	<0.001					258.61	<0.001
Office worker	540	(42.6)	729	(57.4)			179	(27.8)	464	(72.2)		
Self-employed	230	(60.5)	150	(39.5)			95	(67.4)	46	(32.6)		
Part-time worker	89	(54.3)	75	(45.7)			283	(49.0)	294	(51.0)		
Other	19	(65.5)	10	(34.5)			13	(59.1)	9	(40.9)		
Homemaker	11	(52.4)	10	(47.6)			459	(67.0)	226	(33.0)		
Student	12	(38.7)	19	(61.3)			17	(30.9)	38	(69.1)		
Unemployed	220	(55.7)	175	(44.3)			114	(63.0)	67	(37.0)		
Employment status					6.46	0.011					115.43	<0.001
Not working ^c^	243	(54.4)	204	(45.6)			590	(64.1)	331	(35.9)		
Working	878	(47.7)	964	(52.3)			570	(41.2)	813	(58.8)		
Personal annual income, JPY					69.36	<0.001					144.43	<0.001
Less than 2 million yen	267	(59.2)	184	(40.8)			822	(58.8)	575	(41.2)		
2 to 3.99	313	(52.3)	286	(47.7)			153	(32.3)	320	(67.7)		
4 to 5.99	206	(44.1)	261	(55.9)			39	(27.7)	102	(72.3)		
6 to 7.99	77	(30.1)	179	(69.9)			4	(16.7)	20	(83.3)		
8 to 9.99	69	(47.6)	76	(52.4)			7	(43.8)	9	(56.3)		
10 million yen or more	51	(41.8)	71	(58.2)			3	(33.3)	6	(66.7)		
Unknown	136	(55.1)	111	(44.9)			129	(54.0)	110	(46.0)		
Personal annual income ^d^					31.17	<0.001					9.14	0.003
Low	786	(51.8)	731	(48.2)			1014	(50.4)	997	(49.6)		
High	197	(37.7)	326	(62.3)			14	(28.6)	35	(71.4)		
Education level ^e^					0.50	0.481					6.90	0.009
Low	476	(49.8)	479	(50.2)			758	(52.5)	687	(47.5)		
High	645	(48.4)	689	(51.6)			402	(46.8)	457	(53.2)		

* χ^2^ test. ^a^ Age was defined for males as: Low < 53 (median age of males) years and High ≥ 53 years. For females, age was defined as: Low < 48 (median age of females) years and High ≥ 48 years. ^b^ Marital status: Single included those single due to divorce or bereavement. ^c^ Employment status: Not working included Homemaker, Student, and Unemployed. ^d^ Personal income was defined as Low < 6,000,000 JPY and High ≥ 6,000,000 JPY. ^e^ Education level was defined as Low: attended junior high school, high school, or vocational school and High: attended undergraduate studies or above. Abbreviations: HC−, refrained from going for health checkups; HC+, went for health checkups.

**Table 2:** Comparison of the condition of health characteristics in the refrained-from-health-checkups and health-checkups groups by sex.

	Males (n = 2289)						Females (n = 2304)					
Variable	HC−		HC+				HC−		HC+			
	n	(%)	n	(%)	χ^2^	*p* Value *	n	(%)	n	(%)	χ^2^	*p* Value *
Taking medicine (Hypertension)												
					5.66	0.017					4.65	0.031
Yes	194	(43.9)	248	(56.1)			66	(42.0)	91	(58.0)		
No	927	(50.2)	920	(49.8)			1094	(51.0)	1053	(49.0)		
Taking medicine (Diabetes)												
					0.03	0.866					1.00	0.317
Yes	71	(48.3)	76	(51.7)			17	(42.5)	23	(57.5)		
No	1050	(49.0)	1092	(51.0)			1143	(50.5)	1121	(49.5)		
Taking medicine (Hyperlipidemia)												
					18.21	<0.001					7.82	0.005
Yes	98	(36.7)	169	(63.3)			54	(38.8)	85	(61.2)		
No	1023	(50.6)	999	(49.4)			1106	(51.1)	1059	(48.9)		
Skipping breakfast ^a^					27.86	<0.001					9.63	0.002
Yes	317	(58.9)	221	(41.1)			305	(56.2)	238	(43.8)		
No	804	(45.9)	947	(54.1)			855	(48.6)	906	(51.4)		
Physical activity ^b^					10.74	0.001					18.74	<0.001
Yes	402	(44.7)	497	(55.3)			368	(44.3)	462	(55.7)		
No	719	(51.7)	671	(48.3)			792	(53.7)	682	(46.3)		
Walking speed					1.26	0.260					5.77	0.016
Yes	537	(47.8)	587	(52.2)			425	(47.2)	475	(52.8)		
No	584	(50.1)	581	(49.9)			735	(52.4)	669	(47.6)		
Exercise ^c^					10.48	0.001					8.14	0.004
Yes	282	(43.6)	365	(56.4)			164	(43.6)	212	(56.4)		
No	839	(51.1)	803	(48.9)			996	(51.7)	932	(48.3)		
Drinking alcohol ^d^					3.87	0.145					0.94	0.627
Everyday	324	(46.4)	375	(53.6)			156	(49.4)	160	(50.6)		
Sometimes	354	(48.7)	373	(51.3)			336	(49.1)	348	(50.9)		
Rarely drink	443	(51.3)	420	(48.7)			668	(51.2)	636	(48.8)		
Smoking					6.38	0.012					1.23	0.268
Yes	335	(53.3)	294	(46.7)			130	(53.7)	112	(46.3)		
No	786	(47.3)	874	(52.7)			1030	(50.0)	1032	(50.0)		
Increase/decrease in body weight ^e^												
					0.20	0.654					24.45	<0.001
Yes	391	(49.6)	397	(50.4)			479	(57.2)	359	(42.8)		
No	730	(48.6)	771	(51.4)			681	(46.5)	785	(53.5)		
Improve lifestyle					10.89	0.001					0.81	0.369
Yes	760	(46.8)	865	(53.2)			906	(49.9)	911	(50.1)		
No	361	(54.4)	303	(45.6)			254	(52.2)	233	(47.8)		

* χ^2^ test; Questions from a specific health examination questionnaire, partially modified: ^a^ Do you skip breakfast more than three times a week? ^b^ In your daily life, do you walk or do any equivalent amount of physical activity for more than 1 h a day? ^c^ Are you in the habit of doing exercise to sweat lightly for over 30 min at a time, two times weekly, for over a year? ^d^ How often do you drink? (sake, shochu, beer, wine, whisky, brandy, etc.) ^e^ Have you gained or lost more than ±3 kg in the past year? Abbreviations: HC−, refrained from going for health checkups; HC+, went for health checkups.

**Table 3:** Factors associated with health checkups among survey participants, determined by multivariate logistic regression analysis (male).

	Multivariate Analysis		Stepwise		Analysis of Maximum Likelihood Estimates			
Variable	OR	95% CI	OR	95% CI	df	SE	Wald χ^2^	Pr > ChiSq
Age (Low = 1.00)								
High	0.56	0.39–0.80	0.54	0.41–0.71	1	0.180	10.38	0.001
Marital status (Single = 1.00)								
Married	1.05	0.66–1.67	ns		1	0.239	0.036	0.849
Children under 18 years (No = 1.00)								
Yes	1.01	0.72–1.42	ns		1	0.175	0.002	0.963
Employment status (Not working = 1.00)								
Working	1.13	0.77–1.67	ns		1	0.199	0.384	0.536
Personal income (Low = 1.00)								
High	1.75	1.31–2.32	1.80	1.37–2.37	1	0.145	14.79	<0.001
Taking medicine (Hypertension) (Yes = 1.00)								
No	0.71	0.52–0.98	0.71	0.52–0.97	1	0.162	4.464	0.035
Skipping breakfast (Yes = 1.00)								
No	1.69	1.22–2.35	1.73	1.25–2.39	1	0.168	9.708	0.002
Physical activity (Yes = 1.00)								
No	0.82	0.62–1.10	0.75	0.58–0.97	1	0.146	1.797	0.180
Exercise (Yes = 1.00)								
No	0.84	0.61–1.15	ns		1	0.160	1.170	0.279
Smoking (Yes = 1.00)								
No	1.07	0.80–1.42	ns		1	0.147	0.195	0.659
Improve lifestyle (No = 1.00)								
Yes	1.31	0.99–1.73	ns		1	0.143	3.591	0.058

The multivariate logistic regression analysis. Abbreviations: OR, odds ratio; 95% CI, 95% confidence interval; ns, not significant; df, degrees of freedom of a statistic; SE, standard error; Wald χ^2^, Wald test statistic.

**Table 4:** Factors associated with health checkups among survey participants, determined by multivariate logistic regression analysis (Female).

	Multivariate Analysis		Stepwise		Analysis of Maximum Likelihood Estimates			
Variable	OR	95% CI	OR	95% CI	df	SE	Wald χ2	Pr > ChiSq
Marital status (Single = 1.00)								
Married	1.23	0.84–1.79	ns		1	0.193	1.133	0.287
Children under 18 years (No = 1.00)								
Yes	0.68	0.52–0.90	0.66	0.51–0.86	1	0.142	7.119	0.008
Employment status (Not working = 1.00)								
Working	3.08	2.38–3.98	3.02	2.35–3.88	1	0.131	73.52	<0.0001
Personal income (Low = 1.00)								
High	1.38	0.53–3.60	ns		1	0.489	0.436	0.509
Education (Low = 1.00)								
High	0.95	0.73–1.24	ns		1	0.138	0.142	0.706
Taking medicine (Hypertension) (Yes = 1.00)								
No	0.66	0.42–1.04	ns		1	0.231	3.140	0.076
Skipping breakfast (Yes = 1.00)								
No	1.36	0.97–1.90	ns		1	0.171	3.232	0.072
Physical activity (Yes = 1.00)								
No	0.84	0.64–1.11	ns		1	0.139	1.543	0.214
Exercise (Yes = 1.00)								
No	0.76	0.53–1.09	0.68	0.48–0.95	1	0.187	2.216	0.137
Walking speed (Fast = 1.00)								
Slow	0.91	0.69–1.18	ns		1	0.136	0.526	0.469
Increase/decrease in body weight (Yes = 1.00)								
No	1.38	1.06–1.79	1.41	1.09–1.83	1	0.135	5.593	0.018

The multivariate logistic regression analysis. Abbreviations: OR, odds ratio; 95% CI, 95% confidence interval; ns, not significant; df, degrees of freedom of a statistic; SE, standard error; Wald χ^2^, Wald test statistic.

## Data Availability

Due to ethical reasons data are not publicly available.
